# Tailoring educational interventions targeting parental vaccine hesitancy: a systematic review of quantitative studies

**DOI:** 10.3389/fpubh.2026.1793920

**Published:** 2026-04-07

**Authors:** Isa Zappullo, Laura Catalano, Rosalia De Biase, Francesco Panico, Francesca D’Olimpio, Laura Sagliano, Luigi Trojano

**Affiliations:** Department of Psychology, University of Campania Luigi Vanvitelli, Caserta, Italy

**Keywords:** childhood vaccine, health promotion, HPV, intervention, parental vaccine hesitancy, safety promotion, systematic review

## Abstract

**Introduction:**

Childhood vaccines, such as those to prevent tetanus (DTaP/Tdap), human papillomavirus (HPV) or hepatitis B (Hep B), have proven to be an effective strategy in preventing long-term diseases arising from injuries, such as puncture wounds, cuts or burns (e.g., DTaP/Tdap vaccine) or risky sexual behavior in adolescence (e.g., HPV or Hep B vaccines). However, despite national child immunization policies, parental vaccine hesitancy, defined as a delay in accepting or a refusal of available vaccinations, still represents a major social issue, resulting in reduced adherence to numerous vaccination campaigns. Different types of interventions have been proposed to reduce vaccine hesitancy for childhood vaccines, but mixed results have emerged regarding their effectiveness. In the present systematic review, we aimed to perform a qualitative analysis of existing evidence-based interventions targeting parental vaccine hesitancy.

**Methods:**

A systematic search on the PubMed, PsychINFO and Web of Science databases was performed to select the relevant studies, with a timeframe ranging from April 2015 to July 2025. To be included in the study, articles had to focus on educational or psychological interventions to reduce vaccine hesitancy for childhood vaccines, using a quantitative method with pre-post measures to adequately assess the effectiveness of the intervention.

**Results:**

Out of 442 identified articles, 11 studies met selection criteria and were included in this review. All the articles focused on parents as the recipients of the intervention, while the target population of vaccines ranged from infants to adolescents. The most frequently targeted vaccine was for HPV prevention, and intervention durations ranged from a single exposure session to interventions spanning approximately 2 years. The types of intervention can be grouped into four main categories: (i) narrative-based; (ii) web-based; (iii) culturally-targeted; (iv) other communication-based. The review provided qualitative evidence on the effectiveness of interventions targeting parental vaccine hesitancy in each of the identified categories, although with considerable variability.

**Discussion:**

Despite some positive evidence about effectiveness of interventions for promoting immunization in the developmental population, further well-designed evidence-based studies are necessary to reducing heterogeneity of the available data and generalizing conclusions about effective strategies against vaccine hesitancy.

## Introduction

Childhood injuries represent a significant public health concern, not only due to the considerable infant mortality rate, but also in consideration of the long-term consequences of non-fatal conditions (1). Among these, bacterial and viral infections constitute a significant source of morbidity in injured children (2). For instance, in low- and middle-income countries with low immunization rates, life-threatening tetanus often occurs due to burns, wounds and other traumatic injuries (3, 4). In the same vein, early sexual intercourse and other high-risk sexual behaviors in adolescence are associated with an increased incidence of Human Papillomavirus (HPV) and Hepatitis B (HepB) infections, exposing young people to the risk of developing long-term diseases such as cervical and liver cancer, respectively (5, 6).

Childhood vaccines have proven to be an effective strategy in preventing long-term diseases arising from injuries, such as puncture wounds, cuts or burns [e.g., Diphtheria-tetanus-pertussis vaccine—DTaP/Tdap; (7)] or risky sexual behavior in adolescence [e.g., HPV or HepB vaccines; (8, 9)]. However, following the COVID-19 pandemic, a reduction in routine childhood vaccination rates has been observed in several countries (10–13). Far from being exclusively related to low- and middle-income countries, this global phenomenon, well-known as *vaccine hesitancy*, also affects upper-middle-income countries, with the United States and Europe experiencing a dramatic increase in mistrust of vaccines (14, 15). It has been estimated, for example, that more than one-third of US children are not following the early childhood immunization national schedule (16). Despite the plethora of definitions of “vaccine hesitancy,” one of the earliest operationalization of the construct was developed by the SAGE Working Group on Vaccine Hesitancy (17), which defined it as *a delay in acceptance or refusal of vaccination despite availability of vaccination services*. According to the Working Group, vaccine hesitancy represents a multifaceted phenomenon that appears to be context-specific, varying according to time, place and type of vaccine (17).

Consistent with the widely accepted “3 Cs” model, the acceptance of vaccination is the behavioral outcome of a complex decision-making process that can potentially be influenced by three factors: (i) confidence (i.e., trust in vaccines and delivery systems), (ii) complacency (i.e., low perceived risk of disease and self-efficacy), and (iii) convenience [i.e., accessibility of services; (17)]. Additionally, vaccine hesitancy has been associated with factors such as socio-cultural (18–20) or psychological characteristics of the decision-maker, including emotion regulation strategies and personality traits (21). In the context of parental vaccine hesitancy, mis- and disinformation, as well as a lack of clear recommendations from pediatricians, appear to be pivotal in parents’ decision-making process (22–24). Emblematic is the widespread dissemination of fake news, sometimes supported by research misconducts, regarding the link between vaccines and autism, which fuels skepticism and mistrust of vaccines (25, 26). Furthermore, cultural factors, such as morality and religiosity, can contribute to parents’ refusal to vaccinate their children against sexually transmitted infections, such as HPV (27). Indeed, although some factors represent barriers shared with other vaccines [e.g., perception of risks and limited benefits; (28)], prejudice linked to sexually transmissible infections, discussing sex with one’s child, as well as the idea that HPV vaccine could promote inappropriate or unprotected sexual behavior, have been shown to be specific barriers to adolescent vaccination against sexually transmitted diseases (29–34).

A wide range of interventions has been proposed to enhance vaccination coverage rates, including implementation of reminder/recall systems, provision of incentives, and inception of school immunization policies (35, 36). However, these strategies do not specifically address the issue of vaccine hesitancy, which encompasses not only behavioral (i.e., vaccination behavior) but also cognitive and affective aspects [i.e., the psychological state of indecisiveness that people may experience when deciding on vaccination; (37)]. Rather, the most common interventions to reduce vaccine hesitancy are grounded in educational approaches, with the objective of enhancing knowledge about vaccination (38).

Previous relevant systematic reviews on interventions aimed at addressing parental vaccine hesitancy reported the employment of a broad spectrum of strategies, encompassing tailored or untailored educational materials and narratives, delivered through in-person or web-based methods (34, 36, 39–41). However, evidence on the effectiveness of interventions was conflicting, thus hindering the establishment of clear guidelines for implementing evidence-based interventions targeting parental vaccine hesitancy (34, 36, 38, 40). Nonetheless, according to a recent systematic review (41) communication interventions, especially those in person and interactive, are often successful in reducing parental vaccine hesitancy toward childhood vaccinations (i.e., targeted parents of young children up to the age of six). However, this previous review did not target other vaccines typically administered in adolescence (e.g., HPV) and included studies assessing effectiveness of the intervention without a pre-post measurement (i.e., based on a single measurement after the intervention), thus its conclusions appear to be limited in generalizability and robustness.

Therefore, the present systematic review aimed to bridge this gap by performing a qualitative analysis of the available evidence-based interventions addressing vaccine hesitancy toward childhood vaccination, ranging from infants to adolescents. Relevantly here, taking advantage of previous evidence suggesting a more rigorous evaluation of the effectiveness of interventions on vaccine hesitancy (38), the present review strictly focused on educational and psychological interventions adopting quantitative, validated and/or well described pre-post intervention measures to assess parental vaccine hesitancy.

## Methods

The method used in this systematic review was based on the guidelines suggested in the Preferred Reporting Items for Systematic Reviews and Meta-Analyses (PRISMA) initiative^1^ for bibliographic research and data communication in systematic reviews (42).

The present study was not pre-registered. A detailed assessment of the systematic review was carried out using the PRISMA 2020 checklist [(43); see Supplementary Table S3].

*Eligibility criteria.* Studies were considered eligible if they: (i) assessed vaccine hesitancy in humans, targeting population involved in childhood vaccination (e.g., parents or guardians, physicians and minors); (ii) focused on educational or psychological interventions to reduce vaccine hesitancy toward childhood vaccination; (iii) adopted validated and/or well described pre-post intervention measures to assess vaccine hesitancy; (iv) used quantitative research methods; (v) were published in peer-reviewed journals in English language. Exclusion criteria were: (i) studies involving general adult population (i.e., no parents); (ii) interventions addressing related but not specific aspects of vaccine hesitancy (e.g., interventions aimed at reducing immunization pain or targeting vaccine literacy or vaccine advocacy); (iii) immunization campaigns that did not include educational or psychological interventions to reduce vaccine hesitancy toward childhood vaccination (e.g., reminder/recall systems, incentives or school immunization policies); (iv) narrative reviews without original data, conference abstracts, unpublished reports, and doctoral theses.

*Search strategy.* A systematic search was performed on the databases PubMed, PsychINFO and Web of Science to select the relevant studies. Studies conducted from 17 April 2015 to 3 July 2025 were considered in the search strategy. The start of the study sampling timeframe was selected based on the publication of one of the first works aimed at operationalizing the construct of “vaccine hesitancy” by the SAGE Working Group on Vaccine Hesitancy (17), to the aim of enhancing control over the construct under investigation, and in accordance with the recommendation about a more rigorous evaluation of the effectiveness of interventions on vaccine hesitancy (38). The following search string was used and adapted to the specific databases: (intervention OR “educational intervention”) AND (vaccination OR vaccines) AND (refusal OR opposition OR hesitancy OR reluctance OR rejection OR non-adherence OR “non adherence” OR resistance OR skepticism) AND (“psychological antecedents” OR “psychological antecedent” OR “psychological predictors” OR “psychological predictor” OR personality OR obsess* OR paranoi* OR phobi* OR emotion* OR anxiety OR “health anxiety” OR fear OR mood OR worry OR belief* OR “locus of control” OR self-efficacy OR “self efficacy” OR stress OR self-regulation OR “self regulation” OR moral* OR responsibility OR optimism OR “risk perception”) AND (child* OR teenage* OR adolescent* OR parent* OR caregiver* OR mother* OR father* OR school* OR son OR daughter*).

**Figure 1:**
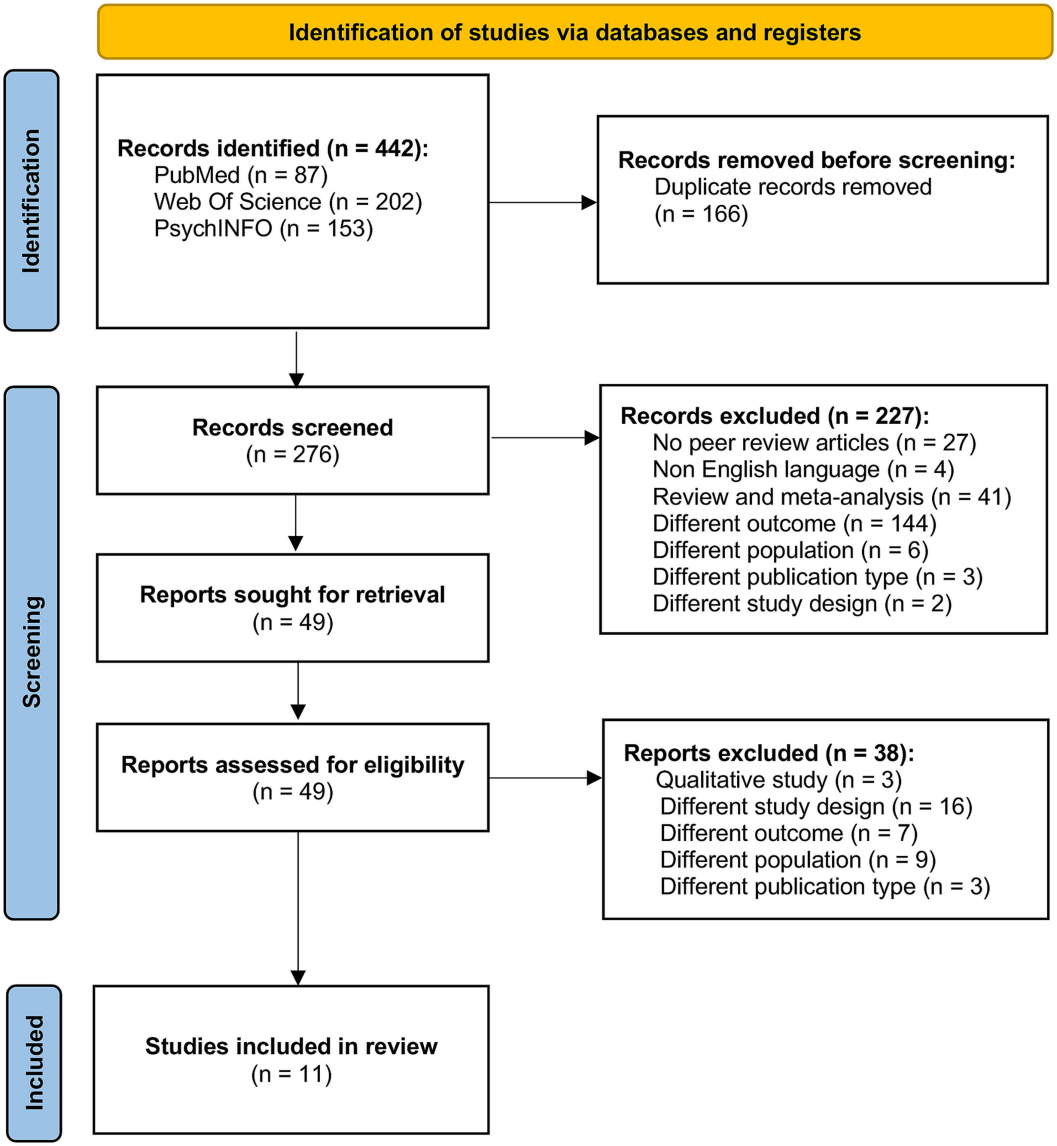
Flow-chart of study selection based on the PRISMA statement. Studies were identified through an advanced search on multiple databases (i.e., PubMed, PsychINFO and Web of Science).

*Studies selection process.* The process of study selection is reported in the PRISMA diagram (Figure 1). Retrieved studies were imported and screened for duplicates by automated tool [Rayyan^2^; also see (44)]. Then, articles were screened by title and abstract to determine their relevance to the study variables. The full texts of the included studies were analyzed to extract the relevant information, and finally the selected studies were rated according to their quality. For each study, the selection, screening and data extraction process, as well as the quality assessment (see below), was performed by two independent researchers (LC and RDB), and any discrepancy was addressed by a third reviewer (FP).

*Data extraction.* The following data were extracted from the included articles: (i) authors and year of publication; (ii) study design; (iii) sample characteristics; (iv) recipients; (v) target vaccination; (vi) characteristics and type of intervention, (vii) pre-post measures of vaccine hesitancy used for assessing the effectiveness of the intervention; (viii) main results.

*Quality assessment.* An adapted version of the Newcastle-Ottawa Scale [NOS; (45)] was used to assess quality and risk of bias of the selected studies (see Supplementary Table S1). The original version of the scale is frequently used to assess the quality of studies included in a systematic review, evaluating the appropriateness of sample selection and outcomes, and to ascertain the comparability of cohorts based on design or analysis. The NOS employs a star scoring system to classify studies as “very good” (9–10 stars), “good” (7–8 stars), “satisfactory” (5–6 stars) and “unsatisfactory” (0–4 stars). In the present study, the original items were modified by incorporating an evaluation of the following: (i) sample size calculation; (ii) pre- and post-intervention assessment of vaccine hesitancy; (iii) the adopted blinding procedure; (iv) the possible confounding factors affecting the data; (v) the adequacy of the statistical approach.

## Results

After checking for duplicates, we excluded 166 of the 442 articles from the primary search. Thus, 276 studies were screened, of which 227 were excluded as they assessed different outcomes or populations or were reviews or meta-analyses. Then, 49 studies were assessed for eligibility and the final study sample included 11 papers (Figure 1). The main characteristics of the included studies were reported in Table 1.

**Table 1:** Key features of the included studies.

Study	Design	Sample	Recipients	Target vaccination	Intervention	Pre-post vaccine hesitancy measure	Main results	NOS-quality assessment
Daley et al. (54)	web-based randomized controlled study	*N* = 1,093 mothers (from high school or less to graduate), randomized into three groups: (i) Social-media Arm (*N* = 542; M age = 31.6; SD = 4.4); (ii) Information Arm (*N* = 371; M age = 31.5, SD = 4.3); (iii) Usual Care Arm (*N* = 180; M age = 31.4; SD = 4.1)	mothers	early childhood vaccine	3 conditions: (i) Vaccine social media arm: access to a website with vaccine information as well as interactive social media components; (ii) Vaccine information arm: access to a website with vaccine information but without social media components; (iii) Usual care arm: routine pediatric preventive care	PACV, ad-hoc survey assessing vaccination benefits, vaccination risks, and perceived self-efficacy at three timepoints (baseline, Timepoint 1 and 2)	Comparing baseline with Timepoint 1 among vaccine-hesitant parents, the intervention arms were associated with significant improvements in attitudes regarding vaccination benefits compared to usual care. Comparing baseline with Timepoint 2 among hesitant parents, the intervention arms were also associated with significant reductions in parental concerns about vaccination risks compared to usual care. No intervention effect was observed among parents not vaccine-hesitant at baseline	3*—unsatisfactory
Dube et al. (46)	online randomized controlled study	*N* = 2000 parents (1,367 females, 18–60 + years, from elementary to graduate) of at least one child aged 5 or younger, randomly assigned into four groups of 500 participants	parents	generic childhood vaccine	Four video conditions (2–4 min): (i) Parents’ informed decision- making; (ii) A mother’s story; (iii) A pediatrician’s story; (iv) A control video on physical exercise	6-point *ad hoc* scale assessing intention to accept future routine vaccine (Routine vaccines intention); 5C Short Scale assessing vaccine attitudes/hesitancy	Having been assigned to any intervention group did not increase participants’ intention to accept future routine vaccines when compared to the controls. A slight but significant difference was observed in all intervention groups, except in the control, on the effectiveness of the narratives to change parents’ attitudes toward vaccination (5C Short Scale)	5*—satisfactory
Gerend et al. (47)	online randomized controlled study	948 (482 females; M age = 41.5; SD = 7.0) US parents/guardians with an unvaccinated HPV child aged 9–17 years, randomly assigned to watch one of four videos: (i) non-narrative informational control (*N* = 236; M age = 40.1; SD = 6.5); (ii) role model only narrative (*N* = 233; M age = 41.6; SD = 7.0); (iii) precancer survivor narrative (*N* = 240; M age = 41.9; SD = 6.9); (iv) cancer survivor narrative (*N* = 239; M age = 41.6; SD = 7.6)	parents	HPV	Participants were randomly assigned to watch one of four videos (ranged about 2–4 min): (i) a non-narrative informational control; (ii) a role model only narrative; (iii) a precancer survivor narrative; (iv) a cancer survivor narrative. All four messages described the health-related consequences of HPV infection and the benefits of HPV vaccination. The control message provided information using a question-and-answer format, while the narrative messages provided information in the context of a story	The primary outcome variable, HPV vaccination intentions, was assessed with three items on a 5-point scale, used in previous research (59). Secondary outcome variables focused on: (i) parent satisfaction with the videos, specifically acceptance [three items adapted from Gerend et al. (74); Kreuter et al. (75)] and rejection [two items used in Witte et al. (76)] of the messages; (ii) emotional engagement [a single item adapted from McQueen et al. (77)]	Participants in the cancer survivor narrative condition reported higher intentions to vaccinate their child within the next year relative to participants in the control condition. Participants in the cancer survivor narrative condition also rated the message as more interesting than participants in the control condition. Results from the hierarchical linear regression analysis predicting vaccination intentions showed significantly higher intentions to vaccinate for participants exposed to the cancer survivor narrative message compared to the control message. Finally, results from the exploratory mediation analysis revealed that emotional engagement was a statistically significant mediator of the effect of the cancer survivor narrative (vs. control) on intentions	6*—satisfactory
Henrikson et al. (55)	clinic-level randomized trial	*N* = 347 mothers of healthy newborns (from <30 to >35 years; from High school or less to Postgraduate), randomly assigned into two groups: control (*N* = 174) and experimental, (*N* = 173) group	mothers	early childhood vaccine	Novel intervention [“Ask, Acknowledge, Advise”; (60)] to address parental hesitancy by improving physician-parent communication about early childhood vaccines. Control clinics did not receive any intervention. The mothers were interviewed at the beginning of the study and after 6 months (phone interview)	PACV	The intervention had no detectable effect on maternal vaccine hesitancy	4*—unsatisfactory
Kwan et al. (56)	web-based randomized controlled trial	*N* = 824 females (from elementary to graduate) in their third trimester of pregnancy or had a child ≤ 2 months of age, randomized in three intervention arms: (i) Usual Care (*N* = 274, M age = 31.8; SD = 4.4); (ii) Untailored Website (*N* = 274; M age = 32.2; SD = 4.2); (iii) Tailored Website (*N* = 276; M age = 32; SD = 4.5)	mothers	early childhood vaccine	3 conditions: (i) Untailored website: included educational content designed to address common beliefs and concerns about childhood vaccines; (ii) Tailored website: included the same content and provided introductory persuasive messages for each page tailored to the individual participant’s stated baseline intention to vaccinate and their vaccination values; (iii) Usual care: no access to the website (78)	Participants completed pre-exposure surveys at baseline (T1), when the infant was between 4 and 6 months (T2), when the infant was between 10 and 12 months (T3), and when the child was between 13 and 15 months of age (T4), including: (i) PACV- short, assessing vaccine hesintancy; (ii) ad-hoc two items scale assessing mothers’ intention to vaccinate their newborn during the first year of life. Following survey completion at time points 1–3, participants assigned to either untailored or tailored website arms were exposed to the website. Vaccination behavior was also collected from electronic medical records	Both tailored and untailored website arms showed similar increases in intention to vaccinate more than usual care. Rates of late vaccination were lowest for those consistently reporting intending to vaccinate all on time between baseline and T3	4*—unsatisfactory
McKeever et al. (48)	online randomized study	*N* = 1,080 US vaccine-hesitant parents (672 females; 18–74 years; 21.7% completed high school; 27.2% completed 4 years of college, while 16.5% completed a postgraduate degree) with at least one child 4 years of age or younger, divided into two intervention groups (Jenny’s First Sleepover; *N* = 550) and control (*N* = 530) condition	parents	early childhood vaccine	“Jenny’s First Sleepover” is a darkly humorous satirical book about childhood vaccinations, designed to influence knowledge, attitudes, and behaviors of vaccine hesitant parents. The booklet utilized in the control condition was a publicly accessible, medically accurate booklet from the CDC encouraging childhood vaccinations	Attitudes toward vaccinations were measured pre- and post test with six bipolar items employing a seven-point response format (1 = Negative to 7 = Positive; 1 = Wrong to 7 = Right; 1 = Foolish to 7 = Wise; 1 = Bad to 7 = Good; 1 = Unfavorable to 7 = Favorable; 1 = Unacceptable to 7 = Acceptable). Also attitude toward the book and negative emotions were evaluated	A direct positive association between the Humorous book condition and Attitudes toward vaccination was found; Homorous book condition was also indirectly related to Attitudes toward vaccination by the mediating effects of Negative emotions and Perceived information quality	3*—unsatisfactory
Olagoke et al. (50)	online randomized controlled trial	*N* = 342 Christian parents (157 females, M age = 41.33; SD = 5.47; from less than collage to college degree or more) of unvaccinated adolescents aged 11–17 years, randomized in intervention (*N* = 175) or control (*N* = 167) group	parents	HPV	HPV Vaccine message with religious content (target intervention) vs. HPV control message	HPV-intention score change (post-pre) based on responses to two ad-hoc items, measured on a five-point Likert scale	The intervention group reported higher and statistically significant intention change that the control group, also controlling for race	4*—unsatisfactory
O'Marr et al. (49)	online randomized trial	*N* = 350 US parents (60% females; from high school to graduate degree) with at least one child under the age of 10 that had not yet begun the HPV vaccine series, randomly assigned to either the intervention group (self- affirmation condition) or the control group (no-affirmation condition; Study 2)	parents	HPV	From a list of 20 values, participants either selected the one most important to them and briefly wrote about why (intervention condition), or selected the value least important to them and wrote about how it could be important for another individual (control condition)	An ad-hoc five-item scale intended to measure their attitudes toward the HPV vaccine as well as their intentions to vaccinate their child	No significant effect of the interaction between the self-affirmation condition and purity on HPV vaccine attitudes and intentions or for the interaction between self-affirmation and loyalty was found. Thus, the affirmation intervention failed to moderate the association between moral foundations and HPV vaccine attitudes	2*—unsatisfactory
Porter et al. (51)	online randomized controlled trial	*N* = 762 parents (537 females; from High School to Graduate or Professional Degree) of females 9–17 years old, randomly assigned into three groups: (i) Centers for Disease Control (CDC) Message (*N* = 271; M age = 39.5; SD = 7.6); (ii) Cervical Cancer Message (*N* = 249; M age = 39.2; SD = 6.6); (iii) Control Message (*N* = 242; M age = 40.2; SD = 7.3)	parents	HPV	3 conditions: (i) standard CDC HPV message; (ii) cervical cancer-salient message; (iii) non-vaccine control message	Questions adapted from the Vaccine Confidence Scale and the PACV-short, assessing HPV vaccination intention and attitudes toward the vaccine	Neither message affected intention to vaccinate or overall vaccine confidence	6*–satisfactory
Redd et al. (52)	online randomized study	1,241 self-identified Christian parents of child under 11 (804 females; 18- > 55 years; from incomplete high school to Post-laureate/professional degree), randomly divided into 3 groups: (i) Control video intervention (*N* = 434 parents, of whom 231 are included in the HPV vaccine hesitant group); (ii) Religious video intervention (*N* = 394 parents, of whom 210 are included in the HPV vaccine hesitant group); (iii) Informational educational video intervention (*N* = 394 parents, of whom 223 are included in the HPV vaccine hesitant group)	parents	HPV	Three intervention videos: (i) Control video intervention: contained information on an adenovirus, but nothing about HPV or vaccines in general; (ii) Religious video intervention: it is a story told by a devout Christian cervical cancer survivor who discussed her experiences and advocated for getting vaccinated; (iii) Informational educational video intervention: contained scientific facts about human papillomaviruses, the diseases they cause, how they are transmitted, and how vaccination protects against them	A validated survey based on Redd et al. (79), including: Beliefs that religious adherence protects against HPV, Intent to vaccinate, Positive attitudes toward vaccines, HPV knowledge, Vaccine knowledge, Religiosity, Pro-vaccine religious views, Religious encouragement of premarital abstinence, Parent/peer influence on sexual behavior, and Trust in modern medicine. 4 items were summed for each participant and used to generate a “HPV Vaccine intention score.” This score was used: (i) to determine effectiveness of the different video interventions on intent to vaccinate against HPV; (ii) to identify participants who were deemed “HPV vaccine hesitant”	The religious-focused and educational interventions significantly improved intentions toward HPV vaccination. The religiously-focused video also significantly diminished the belief that the HPV vaccine is unnecessary because of a family’s values. Parents significantly credited both interventions with improving their intent to vaccinate their children against HPV	3*–unsatisfactory
Woodall et al. (53)	clinic-cluster randomized trial	*N* = 82 parents (92.5% female; M age = 38.96 years; SD = 9.64; from 11th grade or less to Doctorate degree/Other Professional degrees) of an 11–14-year-old daughter who had not yet received HPV vaccination. Three-month follow-up surveys were completed by 38% (*n* = 31) of the study sample (unspecified demographic characteristics)	parents	HPV	A study was conducted on a group of clinics, randomized to receive the Vacteens/Vacunadolescente.org web app or the Usual and Customary (UC) HPV vaccination information (control group).	Parents were assessed by online surveys at baseline and 3-month assessment points. The surveys measured participants’ demographic characteristics, and HPV related variables, including HPV knowledge and HPV vaccine attitudes (e.g., perceived daughters’ risk of HPV, beliefs about HPV and HPV vaccination, intention to have daughter vaccinated, HPV informed decision making). Moreover, Vaccination records were acquired by matching parent identification information to the NM-SIIS database	Parents in the Vacteens.org/Vacunadolescente were more likely to intend to vaccinate their daughters right away than later or not at all. In addition, parents in the Vacteens.org/Vacunadolescente group were significantly more confident about their vaccination choices (Informed Decision Making), and a trend toward being more aware of the benefits and risks of vaccination	3*—unsatisfactory

HPV, Human Papilloma Virus; PACV, Parent Attitudes about Childhood Vaccines. According to the NOS scoring system: Very Good Studies: 9–10*, Good Studies: 7–8*, Satisfactory Studies: 5–6*, Unsatisfactory Studies: 0–4*.

*Quality evaluation of included studies.* Among the 11 studies included in the present systematic review, 8 were classified as unsatisfactory and 3 were classified as satisfactory (Table 1). The major issues in quality evaluation were related to the use of convenience samples, an inadequate between-subject design (i.e., not matched for age, sex, and education), and a lack of justification for the sample size. Additionally, there was inadequate reporting of statistical analyses and control of relevant confounders (see Supplementary Table S2).

*Methods of the selected studies.* All studies involved convenience samples consisting of parents (46–53) or mothers (54–56) as recipients of the intervention, while the target developmental population ranged from infancy to adolescence. The most common targeted vaccination was the vaccine preventing HPV (47, 49–53), while some studies focused on promoting generic (46) or early (48, 54–56) childhood vaccines. The duration of the intervention varied considerably: most interventions were completed in a single exposure session (46–52), whereas only a few adopted a longitudinal approach (53–56). Five of seven single-session studies (46–48, 50, 52), and three of four longitudinal studies (53, 54, 56) found a positive effect of the intervention, showing very similar cumulative percentages between the two methodologies (approximately 71 and 75%, respectively).

In terms of study design, most studies used a randomized (48, 49, 52, 53, 55) or randomized controlled (46, 47, 50, 51, 54, 56) approach. The majority of studies were web-based (54, 56) or conducted online (46–52), while only two were conducted in clinical settings (53, 55).

Finally, about outcome measurement tools, only four of 11 studies used validated measures such as the Parent Attitudes about Childhood Vaccines [PACV; (57)] scale (54–56) or the 5C Short (58) scale (46), whereas the remaining studies used ad-hoc scales adapted from previous studies.

*Detailed evidence description.* Despite the heterogeneity of the studies in terms of characteristics of the intervention, and although some of them combined different approaches, four main categories of intervention could be identified: (i) narrative-based; (ii) web-based; (iii) culturally-targeted; (iv) other communication-based.

*Narrative-based interventions.* Two studies used narrative-based interventions in which participants were shown short videos depicting different actors sharing their personal experiences, so to provide health information and promote positive health behaviors (46, 47). One study involved parents of children aged 5 years or younger to promote general childhood vaccination (46), the other addressed HPV vaccination intentions in parents of children aged between 9 and 17 years (47). In Dube et al.’s study (46), the participants were presented with four video conditions: (i) parents’ informed decision-making, whereby an existing online video shows parents of young children explaining why they finally got their children vaccinated after initially refusing due to online misinformation; (ii) a mother’s story, in which an existing video of a mother and her disabled son shows the consequences of a vaccine-preventable disease; (iii) a pediatrician’s story, namely a newly-developed video in which a pediatrician presents cases of vaccine-preventable diseases encountered in his clinical practice; (iv) a control video of a pediatrician explaining the importance and benefits of physical activity in children. The assessment of vaccine hesitancy was conducted using the 5C Short Scale (58) and an ad-hoc scale designed to evaluate the intention to accept future routine vaccines. There was no significant effect on the parents’ intention to accept routine vaccinations post-intervention. However, a slight but significant difference was observed in all intervention groups, except in the control group, on the effectiveness of the narratives to change parents’ attitudes toward vaccination (5C Short Scale). Similarly, in Gerend et al.’s study (47), the participants were randomly assigned to watch one of four videos describing the health-related consequences of HPV infection and the benefits of HPV vaccine: (i) a non-narrative informational control video, providing information in a question-and-answer format; (ii) a role model narrative video, in which a mother describes her concerns about the HPV vaccine and how she overcame them by talking to her doctor; (iii) a precancer survivor narrative video, in which a mother describes her or her husband’s experience of being diagnosed with precancerous lesions and their subsequent treatment; (iv) a cancer survivor narrative video, in which a mother describes her or her husband’s experience of being diagnosed with different types of cancer and their treatment. HPV vaccination intention was assessed using an ad-hoc scale (59); parent satisfaction with the videos (acceptance and rejection of the messages) and emotional engagement were also evaluated. Participants in the cancer survivor narrative group reported a higher intention to vaccinate their child within the next year and rated the message more interesting than participants in the control group. Results also showed that emotional engagement mediated the effect of the cancer survivor narrative (vs control) on parents’ intentions to vaccinate their children.

*Web-based interventions.* Three studies adopted different types of internet-based educational platforms designed to reduce parental vaccine hesitancy (53, 54, 56). Two three-arm randomized controlled trials (RCTs) investigated the effectiveness of two types of web-based intervention in reducing hesitancy toward early childhood vaccines among pregnant mothers, i.e., a values-tailored intervention (56) and an intervention with interactive social media components (54). In both studies, vaccine hesitancy was evaluated using several measures, among which the Parent Attitudes about Childhood Vaccines (PACV) at different points up to the child reaching approximately 15 months of age. In Daley et al. ‘s study (54), the efficacy of a vaccine social media arm (VSM), incorporating both vaccine information and interactive social media components, was compared with that of a vaccine information arm (VI), which featured only vaccine information without social media components, and a usual care arm. Among vaccine-hesitant parents, both intervention arms (VSM and VI) showed a significant improvement in attitudes toward vaccination benefits at Timepoint 1 (when their child aged 3–5 months) and a significant reduction in parents’ concerns about the risks of vaccination at Timepoint 2 (when their child aged 12–15 months), in comparison to the usual care arm. In the same vein, Kwan et al. (56) compared three conditions: (i) an untailored website, that included educational content aimed at addressing common concerns about childhood vaccines; (ii) a tailored website, that included the same content and provided persuasive messages, tailored to the individual participant’s stated baseline intention to vaccinate and their values with regard to vaccination; (iii) usual care, for which no access to the website was provided. Both tailored and untailored website arms demonstrated similar increases in intention to vaccinate compared to the usual care, with no added benefit of the tailored messaging over the untailored interactive website.

Woodall et al. (53) investigated the effectiveness of a smartphone web app for parents of adolescent girls designed to encourage HPV vaccination. Parents were recruited from pediatric clinics and were randomized to receive the Vacteens/Vacunadolescente.org web app or the Usual and Customary (UC) HPV vaccination information. Although only 38% of the study sample completed the three-month follow-up survey, the results showed that parents in the intervention group were significantly more confident about their vaccination choices and more likely to intend to vaccinate their daughters immediately rather than later or not at all.

*Culturally-targeted interventions.* Three intervention studies aimed at modifying attitudes toward vaccines among specific populations by leveraging social norms, such as religiosity (50, 52) or moral values (49), for addressing the intention to vaccinate against HPV.

One randomized study investigated the effect of religious message in Christian parents of adolescents aged 11 to 17 (50). The religious message contained the same information as the usual care HPV vaccination message (control condition), but, in addition, included a religious metaphor inspired by the story of Noah’s Ark. Results showed that parents who received the religious message reported a higher intention to vaccinate their children than those who received the control one. In another randomized study on Christian parents of children under 11, Redd et al. (52) compared the effectiveness of three intervention videos: (i) religious video, featuring a devout Christian cervical cancer survivor who discussed her experiences and advocated for vaccination; (ii) informational video, containing scientific facts about HPV (e.g., modes of transmission, associated diseases, and vaccine protection); (iii) control video, containing information on an adenovirus, but with no mention of HPV or vaccines in general. The findings indicated that both the religious and informational interventions, but not the control video, significantly improved intentions toward HPV vaccination. Moreover, data demonstrated that the religious video significantly diminished the belief that the HPV vaccine is unnecessary due to a family’s values. O’Marr et al. (49) also verified the effectiveness of an intervention aimed at promoting HPV vaccination among parents of children under the age of 10, but in this case by testing the effect of moral values on parents’ decision-making. From a list of 20 values (e.g., purity), participants were instructed to either select the one most important to them and write a short justification (self-affirmation intervention condition) or select the value least important to them and write about how it could be important for another individual (no-affirmation control condition). No association was found between self-affirmation condition and HPV vaccine attitudes, nor was a significant moderator effect of intervention observed on the relationship between moral values and HPV vaccine attitudes.

*Other communication-based interventions.* This category encompasses a wide range of intervention methods relying on an array of communication strategies to modify vaccination intentions, including messages (51), satirical books (48), and physician communication (55). One randomized study (55) aimed at promoting adherence to early childhood vaccination in mothers of healthy newborns by means a physician-targeted communication intervention [“Ask, Acknowledge, Advise”; (60)] vs. a control condition (no intervention). The Parent Attitudes about Childhood Vaccines (PACV) was administered to mothers via a telephone interview at baseline and 6 months after the beginning of the intervention. Results showed that the intervention had no effect on maternal vaccine hesitancy, nor did it improve physicians’ confidence in addressing parental vaccine hesitancy. In another randomized study on parents of children aged 4 years or younger, Mckeever et al. (48) found that an intervention adopting a darkly humorous satirical strategy led to a significant improvement in attitudes toward childhood vaccination. More in detail, they compared a conventional intervention (i.e., a medically accurate booklet from the Centers for Disease Control, encouraging childhood vaccinations) with another intervention using a darkly humorous satirical book about childhood vaccinations (i.e., “Jenny’s First Sleepover”), which satirically illustrates the effects of vaccine-preventable diseases on unvaccinated children. The results showed a positive association between humorous satirical book condition and attitude toward vaccination, which also appeared to be indirectly associated through the mediating effect of negative emotions and perceived information quality.

Porter et al. (51) investigated the effectiveness of a cervical cancer-salient message, compared with a standard CDC HPV message and a non-vaccine control message, on the HPV vaccination intentions of parents with daughters aged 9–17. Neither message affected intention to vaccinate or overall vaccine confidence.

## Discussion

The present systematic review aimed to provide a comprehensive qualitative analysis of existing evidence-based educational interventions addressing vaccine hesitancy toward childhood vaccination, spanning from infancy to adolescence. Bearing in mind the heterogeneity of the results identified in previous reviews and the calls for more rigorous methods to evaluate intervention effectiveness (34, 36, 38, 40), this study applied stringent inclusion criteria and focused exclusively on studies adopting a quantitative method and utilizing validated and/or well-described pre-post intervention measures for assessing parental vaccine hesitancy.

Overall, the present review reported a broad spectrum of educational interventions, encompassing the use of narratives (46, 47), internet-based educational platforms (53, 54, 56), messages or videos tailored to specific cultural aspects (49, 50, 52) or not (51), and other communication strategies [e.g., use of humor or physician communication; (48, 55)]. These findings add to the already reported multitude of educational interventions implemented to address parental vaccine hesitancy (34, 36, 39–41). All recipients of the selected studies were parents (both or only mothers) and the most used approach was parent-centered educational intervention, in line with previous systematic reviews (36), except for a single study adopting a physician-targeted communication approach (55).

The studies included in the present systematic review most frequently targeted interventions for HPV vaccination. This result could be explained as an effect of the “Global strategy to accelerate the elimination of cervical cancer as a public health problem” (61), which calls on national policies to implement customized strategic actions to achieve the “90 target” (i.e., 90% of girls fully vaccinated with HPV vaccine by age 15 years) of HPV vaccination by 2030.

In terms of outcome measurement tools, most of the 11 selected studies utilized *ad hoc* scales adapted from previous studies, while only four (46, 54–56) employed validated and standardized questionnaires, with the Parent Attitudes about Childhood Vaccines [PACV; (57)] being the most widely used. This finding is consistent with the conclusions of a previous review, suggesting use of PACV to increase the comparability of results across future studies (62).

Eight of the studies (46–48, 50, 52–54, 56) revealed significant effectiveness of the interventions implemented. This finding showed that, even when the analysis is extended to the adolescent population and more stringent and rigorous inclusion criteria are employed, positive effects of interventions for promoting immunization in the developmental population are detectable, in line with a recent review (41). However, it should be noted that half (46, 52, 54, 56) of the eight studies mentioned above found only a non-specific effect of the intervention conditions compared to the control condition. For instance, Daley et al. (54) found that, among vaccine-hesitant parents, both intervention arms (i.e., the VSM, incorporating both vaccine information and interactive social media components, and the VI, including only vaccine information without social media components) showed a significant improvement in attitudes toward vaccination benefits and a significant reduction in parents’ concerns about the risks of vaccination, in comparison to the control (usual care) arm. In the same vein, in Redd et al.’s (52) study showed that both the religious (i.e., a video in which a Christian cervical cancer survivor shared her experience and provided information on the importance of vaccination) and informational (i.e., a video containing scientific facts about HPV) interventions, but not the control video, significantly improved intentions toward HPV vaccination. Therefore, it could be speculated that, far from being a specific effect of the different types of intervention (e.g., web-based or culturally-targeted), the change in vaccine hesitancy measures could be ascribed to availability of more information about vaccination. This view is consistent with a recent study showing that the need for further information is a significant predictor of parental vaccine refusal (63), thus providing renewed evidence in support of the classic “knowledge-deficit” approach, whereby vaccine hesitant individuals would change their minds if they received the appropriate information [see (38)].

Each of the other four studies reporting a significant effect on intervention was classified in a different category of intervention identified here, providing evidence of effectiveness for narrative-based (47), web-based (53), culturally-targeted (50) and other communication-based (48) interventions. However, only one (47) among these four studies met the criteria for methodological adequacy. In this study, Gerend et al. (47) compared the effectiveness of three narrative videos (i.e, a role model narrative video, a precancer survivor narrative video and a cancer survivor narrative video) with a non-narrative informational control video, observing that only participants in the cancer survivor narrative group reported a higher intention to vaccinate their children within the next year compared to participants in the control group, an effect significantly mediated by emotional engagement. Previous reviews and meta-analyses have suggested that narrative approaches are emerging as a promising set of tools for motivating and supporting health-behavior change (64) and that, compared to statistical (informational) evidence, narrative evidence has a stronger influence on the intention to adopt healthy behavior (65). The results of Gerend et al. (47) lend support to the idea that, beyond “knowledge deficit,” vaccine hesitancy involves many other factors including emotional aspects [see (21, 37, 38)]. Therefore, the findings of this systematic review, although not sufficient for providing solid recommendations, underscore the necessity for additional well-designed studies to enable a rigorous comparison of the effectiveness of narrative-based intervention approaches with other methodologies (e.g., web-based, culturally tailored, and other communication-based approaches).

In the studies selected for the present review the percentage of those reporting a significant effect of the intervention was very similar among those with a longitudinal approach [i.e., three out of four longitudinal studies; (53, 54, 56)] and those with a single-session approach [i.e., five out of seven single-session studies; (46–48, 50, 52)]. The lack of relevant differences between the two approaches is observed even when considering only studies with adequate methodological quality [i.e., none of four longitudinal studies; two of seven single-session studies, (46, 47)], or only studies that identified a specific effect of the experimental intervention condition [i.e., one of four longitudinal studies, (53); three of seven single-session studies, (47, 48, 50)]. This finding is consistent with a previous review showing that one-time interventions were successful in improving childhood immunization and related outcomes such as maternal knowledge of immunization (66).

A further finding to be discussed is related to the quality of the included studies. Indeed, most of the included studies (8 out of 11) failed to meet satisfactory quality criteria, thus limiting the generalizability of the current evidence. The prevalence of convenience sampling and the frequent omission of sample size justifications suggest a high risk of selection bias. Furthermore, the inconsistent control for key demographic variables (age, sex and education) and for confounders represent significant methodological gaps that may mask the true efficacy of the educational and psychological interventions examined.

Some limitations of the present review merit comment. First, while the inclusion criteria adopted here respond to a specific need for a more rigorous assessment of the effectiveness of interventions on vaccine hesitancy (34, 36, 38, 40), this may have contributed to a potential underestimation of the study sample. The present review predominantly comprised studies conducted online or with web-based approach, precluding an adequate comparative analysis with in-person studies. In the same vein, a meta-analytical approach could not be adopted because of the low number of interventions similar enough to be categorized together, insufficient to demonstrate effectiveness using recognized validation criteria [see (38)], and because of the high variability of outcomes across studies (36). Furthermore, as in some previous reviews (36, 40), most of the selected studies were conducted on US population, thereby undermining the generalizability of the conclusions, especially regarding low- and middle-income countries. Other factors, such as bias in language selection (i.e., English language as an inclusion criterion) as well as the prevalence of studies addressing HPV vaccine, may have reduced the generalizability of our findings. As recalled above, vaccine hesitancy is a multifaceted phenomenon which appears to be context-specific, varying as a function of a multitude of factors, including the type of vaccine (17). In particular, the reluctance to vaccinate children against sexually transmitted diseases such as HPV, although sharing common barriers with other vaccines (e.g., risk perception), appears to be specifically related to cultural factors, such as morality and religiosity, with a potential impact on the communication strategies identified to contrast the phenomenon (27–34). Therefore, the paucity of studies addressing early and generic childhood vaccines has potentially narrowed the spectrum of recommendations and prevention strategies identified in this review. In this regard, as we only selected interventions addressing specific aspects of vaccine hesitancy, it should be noted that interventions aimed at reducing relevant aspects associated with, but not overlapping with, vaccine hesitancy, such as those addressing immunization pain [e.g., see (67)] or vaccine literacy or advocacy [e.g., see (68)] were not included in our review and, therefore, call for specific future investigation. Finally, a remark is necessary on the high risk of bias observed in the reviewed studies, which could have potentially affected the conclusions. As stated above, previous relevant systematic reviews have reported a lack of good-quality evidence on the effectiveness of interventions addressing parental vaccine hesitancy, hindering the development of evidence-based guidelines (34, 36, 38, 40). The present synthesis of the literature concurs with these observations. While the results indicated encouraging findings of overall effectiveness of educational interventions aimed at reducing parental vaccine hesitancy, the evidence of good quality was limited and inconsistent, precluding the development of recommendations regarding the specific evidence-based approach to use.

A conclusive consideration is needed on the relationship between vaccines and child injuries. Vaccines are included in the prophylaxis of the most common childhood injuries [e.g., animal bites; (69)] and have demonstrated proven efficacy in preventing long-term diseases resulting from them [e.g., DTaP/Tdap and HPV; (7–9)]. Although the harmful effects of initially mild injuries in unvaccinated children have been well documented [e.g., (70–72)], there is a lack in the literature of studies estimating the specific effect of vaccination in reducing the consequences of child injuries [not only the direct health benefits, but also in micro- and macro-economic terms; see (73)].

Notwithstanding the above limitations, the present systematic review provides a comprehensive synthesis of existing evidence-based educational interventions addressing parental vaccine hesitancy. The findings call for further well-designed studies to facilitate a rigorous comparison of the effectiveness of different kinds of educational intervention (e.g., narrative-based, web-based, culturally-tailored and other communication-based approaches) and guide the implementation of evidence-based public health strategies to promote immunization in the developmental population. The development of best practices for managing parental vaccine hesitancy could be a complementary strategy in the primary prevention of the long-term consequences of non-fatal childhood injuries, especially in low- and middle-income countries with low immunization rates.

## Data Availability

The original contributions presented in the study are included in the article/Supplementary material, further inquiries can be directed to the corresponding author.

## Footnotes

1
https://www.prisma-statement.org/

2
https://www.rayyan.ai
