# Supplementary material for: Tailoring educational interventions targeting parental vaccine hesitancy: a systematic review of quantitative studies
Source: Front Public Health. 2026 Apr 7;14:1793920. doi: 10.3389/fpubh.2026.1793920 (PMC13096038; doi:10.3389/fpubh.2026.1793920)
Supplement: Supplementary file 1 [file Table_1.docx]

**Supplementary materials**

**Table S1.** Newcastle-Ottawa Scale (NOS; Wells et al., 2000) adapted for the present systematic review

**Selection - Maximum 6 stars**

*Representativeness of the sample*:

a) Truly representative of the target population *

b) Somewhat representative of the target population *

c) Selected group of users or convenience sample

d) No description of the sampling strategy

*Sample size:*

a) Satisfactory a-priori power analysis **

b) Satisfactory post-hoc power analysis *

c) Not justified or satisfactory or no information provided

*Selection of the controls*:

a) Within-between-subject studies: matched sample for age, sex, education *

b) Within-subject studies or not matched within-between-subject studies

*Measurement of pre-post vaccine hesitancy variable:*

a) Validated measurement tool **

b) Non-validated measurement tool, but the tool is available or described *

c) No description of the measurement tool

**Comparability - Maximum 1 star**

*Confounding factors controlled:*

a) Data/results adjusted for relevant predictors/risk factors/confounders (education, age and sex) *

b) Data/results not adjusted for all relevant confounders/risk factors/information not provided

**Outcome - Maximum 3 stars**

*Assessment of the outcome:*

a) Double blind assessment **

b) Single blind *

c) No description

*Statistical test:*

a) The statistical test used to analyze the data is clearly described and appropriate, and the measurement of the association (including confidence intervals or: effect size, non-significant effects; all value of statistic methods; mean and SD or ES) are presented **

b) Tests are appropriate, there is p value all value of statistic methods, but reporting is incomplete *

c) The statistical test is not appropriate or not described

**TOTAL**

a) Very Good Studies: 9-10 stars

b) Good Studies: 7-8 stars

c) Satisfactory Studies: 5-6 stars

d) Unsatisfactory Studies: 0 to 4 stars

**Table S2**. Summary assessment of included studies based on the NOS

| **Study** | **Design** | **Selection** | | | | **Comparability** | **Outcome** | | **Total** |
| --- | --- | --- | --- | --- | --- | --- | --- | --- | --- |
|  |  | *Representativeness* | *Sample size* | *Controls* | *Measures* | *Confounding* | *Blinding* | *Statistics* |  |
| Daley et al., 2018 | web-based randomized controlled study | - | - | - | ** | - | - | * | 3* |
| Dube et al., 2024 | online randomized controlled study | - | ** | - | ** | - | - | * | 5* |
| Gerend et al., 2025 | online randomized controlled study | - | ** | * | * | - | * | * | 6* |
| Henrikson et al., 2015 | clinic-level randomized trial | - | - | - | ** | - | * | * | 4* |
| Kwan et al., 2022 | web-based randomized controlled trial | - | - | - | ** | - | - | ** | 4* |
| Mckeever et al., 2025 | online randomized study | - | - | - | * | - | - | ** | 3* |
| Olagoke et al., 2023 | online randomized controlled trial | - | - | - | * | - | ** | * | 4* |
| O'Marr et al., 2023 | online randomized trial | - | * | - | * | - | - | - | 2* |
| Porter et al., 2018 | online randomized controlled trial | - | ** | * | * | * | - | * | 6* |
| Redd et al., 2024 | online randomized study | - | * | - | * | - | - | * | 3* |
| Woodall et al., 2021 | clinic-cluster randomized trial | - | - | - | * | - | * | * | 3* |

***Note*.** According to the NOS scoring system: Very Good Studies: 9-10 *, Good Studies: 7-8 *, Satisfactory Studies: 5-6 *, Unsatisfactory Studies: 0-4 *

**Table S3.** PRISMA 2020 checklist (Page et al., 2021)

| **Section and Topic** | **Item #** | **Checklist item** | **Location where item is reported** |
| --- | --- | --- | --- |
| **TITLE** | | |  |
| Title | 1 | Identify the report as a systematic review. | Title |
| **ABSTRACT** | | |  |
| Abstract | 2 | See the PRISMA 2020 for Abstracts checklist. | Abstract |
| **INTRODUCTION** | | |  |
| Rationale | 3 | Describe the rationale for the review in the context of existing knowledge. | Introduction |
| Objectives | 4 | Provide an explicit statement of the objective(s) or question(s) the review addresses. | Introduction |
| **METHODS** | | |  |
| Eligibility criteria | 5 | Specify the inclusion and exclusion criteria for the review and how studies were grouped for the syntheses. | Methods – “Eligibility criteria” section |
| Information sources | 6 | Specify all databases, registers, websites, organisations, reference lists and other sources searched or consulted to identify studies. Specify the date when each source was last searched or consulted. | Methods – “Search strategy” section |
| Search strategy | 7 | Present the full search strategies for all databases, registers and websites, including any filters and limits used. | Methods – “Search strategy” section |
| Selection process | 8 | Specify the methods used to decide whether a study met the inclusion criteria of the review, including how many reviewers screened each record and each report retrieved, whether they worked independently, and if applicable, details of automation tools used in the process. | Methods – “Studies selection process” section |
| Data collection process | 9 | Specify the methods used to collect data from reports, including how many reviewers collected data from each report, whether they worked independently, any processes for obtaining or confirming data from study investigators, and if applicable, details of automation tools used in the process. | Methods – “Quality assessment” section |
| Data items | 10a | List and define all outcomes for which data were sought. Specify whether all results that were compatible with each outcome domain in each study were sought (e.g. for all measures, time points, analyses), and if not, the methods used to decide which results to collect. | Methods – “Data extraction” section |
|  | 10b | List and define all other variables for which data were sought (e.g. participant and intervention characteristics, funding sources). Describe any assumptions made about any missing or unclear information. | Methods – “Data extraction” section |
| Study risk of bias assessment | 11 | Specify the methods used to assess risk of bias in the included studies, including details of the tool(s) used, how many reviewers assessed each study and whether they worked independently, and if applicable, details of automation tools used in the process. | Methods – “Quality assessment” section |
| Effect measures | 12 | Specify for each outcome the effect measure(s) (e.g. risk ratio, mean difference) used in the synthesis or presentation of results. | Not reported |
| Synthesis methods | 13a | Describe the processes used to decide which studies were eligible for each synthesis (e.g. tabulating the study intervention characteristics and comparing against the planned groups for each synthesis (item #5)). | Methods – “Data extraction” section |
|  | 13b | Describe any methods required to prepare the data for presentation or synthesis, such as handling of missing summary statistics, or data conversions. | Not reported |
|  | 13c | Describe any methods used to tabulate or visually display results of individual studies and syntheses. | Methods – “Data extraction” section |
|  | 13d | Describe any methods used to synthesize results and provide a rationale for the choice(s). If meta-analysis was performed, describe the model(s), method(s) to identify the presence and extent of statistical heterogeneity, and software package(s) used. | Results – “Detailed evidence description” section |
|  | 13e | Describe any methods used to explore possible causes of heterogeneity among study results (e.g. subgroup analysis, meta-regression). | Not applicable |
|  | 13f | Describe any sensitivity analyses conducted to assess robustness of the synthesized results. | Not applicable |
| Reporting bias assessment | 14 | Describe any methods used to assess risk of bias due to missing results in a synthesis (arising from reporting biases). | Supplementary materials - Table S1 |
| Certainty assessment | 15 | Describe any methods used to assess certainty (or confidence) in the body of evidence for an outcome. | Not reported |
| **RESULTS** | | |  |
| Study selection | 16a | Describe the results of the search and selection process, from the number of records identified in the search to the number of studies included in the review, ideally using a flow diagram. | Results – Figure 1 |
|  | 16b | Cite studies that might appear to meet the inclusion criteria, but which were excluded, and explain why they were excluded. | Results – Table 1 |
| Study characteristics | 17 | Cite each included study and present its characteristics. | Results – “Methods of the selected studies” and “Detailed evidence description” sections |
| Risk of bias in studies | 18 | Present assessments of risk of bias for each included study. | Results – “Quality evaluation of included studies” section; Supplementary materials - Table S2 |
| Results of individual studies | 19 | For all outcomes, present, for each study: (a) summary statistics for each group (where appropriate) and (b) an effect estimate and its precision (e.g. confidence/credible interval), ideally using structured tables or plots. | Not reported |
| Results of syntheses | 20a | For each synthesis, briefly summarise the characteristics and risk of bias among contributing studies. | Results – “Quality evaluation of included studies” “Detailed evidence description” sections; Supplementary materials - Table S2 |
|  | 20b | Present results of all statistical syntheses conducted. If meta-analysis was done, present for each the summary estimate and its precision (e.g. confidence/credible interval) and measures of statistical heterogeneity. If comparing groups, describe the direction of the effect. | Results – Table 1 |
|  | 20c | Present results of all investigations of possible causes of heterogeneity among study results. | Not reported |
|  | 20d | Present results of all sensitivity analyses conducted to assess the robustness of the synthesized results. | Not applicable |
| Reporting biases | 21 | Present assessments of risk of bias due to missing results (arising from reporting biases) for each synthesis assessed. | Supplementary materials - Table S2 |
| Certainty of evidence | 22 | Present assessments of certainty (or confidence) in the body of evidence for each outcome assessed. | Not reported |
| **DISCUSSION** | | |  |
| Discussion | 23a | Provide a general interpretation of the results in the context of other evidence. | Discussion |
|  | 23b | Discuss any limitations of the evidence included in the review. | Discussion |
|  | 23c | Discuss any limitations of the review processes used. | Discussion |
|  | 23d | Discuss implications of the results for practice, policy, and future research. | Discussion |
| **OTHER INFORMATION** | | |  |
| Registration and protocol | 24a | Provide registration information for the review, including register name and registration number, or state that the review was not registered. | Methods (statement that the review was not registered) |
|  | 24b | Indicate where the review protocol can be accessed, or state that a protocol was not prepared. | Not reported |
|  | 24c | Describe and explain any amendments to information provided at registration or in the protocol. | Not reported |
| Support | 25 | Describe sources of financial or non-financial support for the review, and the role of the funders or sponsors in the review. | “Funding information” and “Funding statement” sections |
| Competing interests | 26 | Declare any competing interests of review authors. | “Conflict of interest statement” section |
| Availability of data, code and other materials | 27 | Report which of the following are publicly available and where they can be found: template data collection forms; data extracted from included studies; data used for all analyses; analytic code; any other materials used in the review. | “Data availability statement” section |
